# Factors Affecting the Agreement Between Emergency Psychiatrists and General Practitioners Regarding Involuntary Psychiatric Hospitalizations

**DOI:** 10.1038/srep28134

**Published:** 2016-06-21

**Authors:** Pierre Alexis Geoffroy, Alain Duhamel, Hélène Behal, Nadia Zouitina-Lietaert, Julie Duthilleul, Louise Marquette, François Ducrocq, Guillaume Vaiva, Benjamin Rolland

**Affiliations:** 1 Inserm, U1144, Paris, F-75006, France; Université Paris Descartes, UMR-S 1144, Paris, F-75006, France; 2 Université Paris Diderot, Sorbonne Paris Cité, UMR-S 1144 Paris F-75013, France; 3 AP-HP, GH Saint-Louis – Lariboisière – F. Widal, Département de Psychiatrie et de Médecine Addictologique, 75475 Paris Cedex 10, France; 4 Département de Biostatistiques, CHU Lille, Lille, France; 5 Service de Psychiatrie de l’adulte, CHU Lille, Lille, France; 6 Pôle de l’Urgence, CHU Lille, Lille, France; 7 Service d’Addictologie, CHU Lille, Lille, France; 8 SCALab, UMR CNRS 9193, Univ Lille, Lille, France; 9 INSERM U1171, Univ Lille, Lille, France

## Abstract

Important discrepancies exist between physicians in deciding when to perform involuntary hospitalization measures (IHMs). The factors underlying these differences are poorly known.

We conducted a two-year single-center retrospective study in France on patients who were referred to the emergency department (ED) with an IHM certificate written by a private-practice General Practitioner (GP). For each consultation, the official IHM motive was categorized into four groups: Suicide; Psychosis, Mania, or Melancholia (PMM); Agitation; and Other. The alcohol status of the patient was also noted. The factors underlying the ED psychiatrists’ confirmation of the use of IHMs were determined using a logistic regression model. One hundred eighty-nine cases were found (165 patients; 44.2 ± 16 years, 41.3% women). The ED psychiatrists confirmed the use of IHMs in 123 instances (65.1% agreement rate). Multivariate analyses found that IHM disagreement was significantly associated with patient alcohol status and the reason for referral. Specifically, there was an increased risk of IHM disagreement when the patient had an alcohol-positive status (OR = 15.80; 95% CI [6.45–38.67]; p < 0.0001) and when the motive for IHM was “agitation” compared with “suicide” (OR = 11.44; 95% CI[3.38–38.78]; p < 0.0001). These findings reflect significant disparities between GPs and ED psychiatrists regarding the decision to proceed to an IHM.

Involuntary hospitalization measures (IHMs) are legal procedures that impose psychiatric hospitalization on patients with a hazardous ongoing psychiatric disorder who have been clinically assessed as mentally unable to consent to care. In many European countries, any physician can implement an IHM measure^1^. However, some assessment discrepancies have been found between psychiatrists in deciding when to use such measures^2^. Important differences regarding the use of IHMs have also been found between psychiatrists and emergency department (ED) physicians^3^,^4^. However, the agreement between private practice general practitioners (GPs) and ED psychiatrists has not been investigated to date. Moreover, studies addressing whether the clinical profile of a patient can influence the hospitalization decision at the ED have not been conducted regarding IHMs, although these types of analyses have been previously performed for more general situations^5^. Finally, although the issue of assessing alcohol status for IHM is very frequent in clinical practice, this is poorly addressed in the international literature.

We conducted a study in France focusing on IHMs for psychiatric disorders in EDs. We aimed to assess whether ED psychiatrists’ agreement to use the IHMs initiated by private practice GPs depended on i) the clinical referral motive and ii) the alcohol status of the patient.

## Materials and Methods

### Type of study

A retrospective single-center study was conducted using the electronic medical records of the patients who received an emergency psychiatric consultation at the ED of the regional university hospital in Lille, France, between November 2010 and November 2012.

### Data collection

The clinical data were completed by each of the 42 psychiatrists who rotate at the ED. Official documents, including IHM certificates, are systematically included in patient medical records. All of the records of the patients referred to the ED with an IHM certificate were thus examined. Only the subjects whose certificate was written by a private-practice GP were considered for the analyses. The primary clinical motives for requesting an IHM in the certificate were categorized as follows: 1) ‘Suicide’ for suicidal ideation or crisis; 2) psychotic, manic, or melancholic (‘PMM’) symptoms, i.e., delusions, hallucinations, disorganized speech, negative symptoms of schizophrenia, manic symptoms, or melancholic characteristics of a major depressive episode; 3) ‘Agitation’ for agitation, aggressiveness, or violence, excluding categories 1 and 2; and 4) ‘Other’. For cases in which both motives 1 and 2 were provided in the IHM certificate, the subject was categorized as ‘motive 1’.

In the electronic medical records, participant age (years), gender, and alcohol-positivity (defined either by the clinical observation of the ED psychiatrist that the speech or the behavior of the patient was affected by alcohol or by a positive blood alcohol concentration) were also collected.

### Statistical analyses

Age is reported as the mean ± standard deviation. Categorical variables are provided as numbers and percentages.

IHM and non-IHM groups were compared with respect to age using Student t-test and on alcohol status, gender and referral motive using Chi-square tests. A multivariate analysis was performed using logistic regression analysis and included parameters with p < 0.20 in univariate analyses as the independent variables and IHM status as the dependent variable. The odds-ratios for non-IHM were calculated with a 95% CI.

Statistical testing was conducted at the two-tailed α level of 0.05. Data were analyzed using the SAS software package version 9.3 (SAS Institute, Cary, NC, USA).

### Ethical approval

In accordance with French laws, the study’s data extraction and analysis procedures were previously submitted to and approved by the *Commission Nationale de l’Informatique et des Libertés* (Déclaration CNIL # 09112011).

## Results

In total, 4,413 emergency psychiatric consultations were conducted during the studied period. One hundred eighty-nine consultations concerning 165 patients (44.2 ± 16 years, 41.3% women) were referred with an IHM certificate written by a GP (Table 1). The clinical motives provided on the IHM certificates were suicide for n = 99 (52.4%), PMM for n = 51 (27.0%), agitation for n = 30 (15.9%), and other for n = 9 (4.7%). Fifty-five patients (29.1%) were found to be alcohol-positive; of these patients, 31 (56.4%) were referred for an IHM for suicide reasons, 5 (9.1%) for PMM, 18 (32.7%) for agitation, and one for other reasons. The percentage of subjects with positive alcohol status was 31% (n = 31/99) for suicide reasons, 10% (n = 5/51) for PMM, 60% (n = 18/30) for agitation, and 11% (n = 1/9) for others reasons; and so we found a strong relationship between diagnosis categories and positive alcohol status (p < 0.0001).

IHMs were confirmed by the ED psychiatrist for 123 cases (65.1%). Of the 66 cases that did not result in an IHM, 14 of the subjects were permitted to leave the ED after psychiatric consultation, 46 were eventually hospitalized voluntarily in the psychiatric ward, and 6 were hospitalized in non-psychiatric departments. As shown in Table 1, patient alcohol status and GP referral motive were significantly associated with the decision to not proceed to an IHM.

In the multivariate analysis, both alcohol status and reasons for referral remained significantly associated with the decision to not pursue an IHM. In this multivariate analysis, suicidal reasons were considered as the reference category for the motives of referral. Patient positive alcohol status resulted in an increased risk of the ED psychiatrist disagreeing with the GP referral for IHM, as did referrals that were made because of agitation or other motives, both compared with referrals due to risk of suicide (Table 2). We performed a sensitivity analysis which consisted in repeating the multivariate analysis after excluding the 9 patients categorized with other diagnosis, and we found similar multivariate odds ratios (data not shown).

## Discussion

The objective of this study was to determine whether the agreement between ED psychiatrists and GPs regarding IHMs depended on the reason behind the clinical referral and the alcohol status of the patient. Our main and most noteworthy finding was that a patient’s positive alcohol status was associated with a much lower confirmation rate of IHMs. In contrast, the absence of a positive alcohol status was strongly associated with agreement to use an IHM, with very few disagreements. The motive for referral was also associated with the use of an IHM; IHM referrals for agitation and other non-suicidal and non-PMM motives were correlated with an increased likelihood of IHM disagreement compared with referrals for reasons related to suicide. Although we observed a strong relationship between agitation and positive alcohol status, our results demonstrated an independent association of disagreements between GPs and psychiatrists with both agitation and positive alcohol status, since both variables remained significant in the multivariate analysis.

The main limitation of our findings was that this study was only conducted in a single center and only in France, meaning that the results may be skewed by local characteristics. Nevertheless, the exhaustive collection of data across the studied period reduced the possibility of exposure to biases. In addition, the four categories of reasons for referral, which were determined prior to the implementation of the study, were broad categories that did not enable detailed sub-analyses regarding further clinical aspects, e.g., the different types of psychotic disorders or mood disorders.

Despite these limitations, our findings reflect significant disparities between GPs and ED psychiatrists regarding the decision to proceed to an IHM. These differences were primarily related to the alcohol statuses of the patients. GPs and ED psychiatrists were in agreement with a greater increased confirmation of the IHM when patients had a negative alcohol status. Whereas GPs still referred patients for IHMs when they had a positive alcohol status, ED psychiatrists ultimately authorized few of such IHMs. Deciding to use an IHM typically requires an accurate psychiatric evaluation, which is rarely possible in the case of alcohol intoxication. Moreover, an IHM for substance misuse is generally not considered useful and ethical by psychiatrists and addiction specialists^6^,^7^, although this point is still under debate^8^. The differences between GPs and psychiatrists in our study are consistent with previous findings, in which frequent discrepancies existed between psychiatrists and emergency physicians when deciding to resort to an IHM^3^,^4^. Our findings could reflect a conceptual difference between GPs and psychiatrists regarding the IHMs’ objectives, or it could indicate that GPs have less experience with IHMs. This hypothesis concerning GPs’ lack of experience is supported by the fact that several European countries have decided to entrust the performance of IHMs to only previously certified physicians^1^. If confirmed, this would indicate a need for better training of GPs regarding medical objectives of IHMs.

However, anecdotal discussions with GPs who referred alcohol-positive patients with aggressiveness to the ED using IHMs have revealed that, in some cases, the physicians were well aware that the IHM would ultimately not be enforced, but writing an IHM certificate was the only way an ambulance would accept transportation of the patient to the hospital. These cases thus indicate that there may be regulatory issues in referring intoxicated patients with behavioral complications to the ED. Understanding the differences in practice and the issues regarding the conditions underlying IHMs across Europe appears to be an important step in ensuring homogenous ethical and evidence-based clinical decisions regarding the restriction of an individual’s freedom due to medical reasons. Unfortunately, international research has been poorly developed on this topic.

## Additional Information

**How to cite this article**: Geoffroy, P. A. *et al*. Factors Affecting the Agreement Between Emergency Psychiatrists and General Practitioners Regarding Involuntary Psychiatric Hospitalizations. *Sci. Rep.* **6**, 28134; doi: 10.1038/srep28134 (2016).

## Figures and Tables

**Table 1:** Comparisons between the consultations in which psychiatrists agreed with the GP’s IHM referral and those in which the psychiatrist did not agree.

	Whole sample	not-validated IHM	validated IHM	p
Number of consultations	189	66 (34.9%)	123 (65.1%)	
Mean age (years; m ± SD)	44.2 ± 16.0	46.6 ± 16.3	42.9 ± 15.8	0.13
Gender (n females; %)	78 (41.3%)	32 (48.5%)	46 (37.4%)	0.14
Alcohol-positive	55 (29.1%)	42 (63.6%)	13 (10.6%)	<0.0001
IHM motives				<0.0001
1 = suicidal ideation/suicide crisis	99 (52.4%)	30 (45.4%)	69 (56.1%)	
2 = Psychotic/manic or melancholic symptoms	51 (27.0%)	5 (7.6%)	46 (37.4%)	
3 = agitation/aggressiveness (motives 1 & 2 excluded)	30 (15.9%)	24 (36.4%)	6 (4.9%)	
4 = other	9 (4.7%)	7 (10.6%)	2 (1.6%)	

IHM: involuntary hospitalization measure.

**Table 2:** Results of the multivariate model analyzing the factors affecting disagreement between emergency psychiatrists and general practitioners regarding involuntary hospitalization measures.

	Odds Ratio	95% CI	*p*
Age (per 10 year increase)	1.16	0.87 – 1.55	0.31
Gender (female vs. male)	1.75	0.73 – 4.21	0.21
Alcohol status (positive vs. negative)	15.80	6.45 – 38.67	<0.0001
IHM motive			<0.0001*
1 = Suicide (reference)	1	–	–
2 = PMM	0.54	0.16 – 1.80	0.31
3 = Agitation	11.44	3.38 – 38.78	<0.0001
4 = Other	19.19	3.10 – 118.96	<0.002

Suicide = suicidal ideas/crisis, PMM = psychotic, manic, or melancholic symptoms, Agitation = agitation, aggressiveness, or violence, excluding cases 1 or 2.

^*^p for comparison between IHM motive and IHM validated by psychiatrists.
